# Impairments of Social Motor Coordination in Schizophrenia

**DOI:** 10.1371/journal.pone.0029772

**Published:** 2012-01-17

**Authors:** Manuel Varlet, Ludovic Marin, Stéphane Raffard, R. C. Schmidt, Delphine Capdevielle, Jean-Philippe Boulenger, Jonathan Del-Monte, Benoît G. Bardy

**Affiliations:** 1 Movement to Health Laboratory, EuroMov, Montpellier-1 University, Montpellier, France; 2 Epsylon, Laboratory Dynamic of Human Abilities and Health Behaviors, Department of Sport Sciences, Psychology and Medicine, University of Montpellier and St-Etienne, France; 3 University Department of Adult Psychiatry, CHU Montpellier, Montpellier-1 University, Hôpital de la Colombière, Montpellier, France; 4 Department of Psychology, College of the Holy Cross, Worcester, Massachusetts, United States of America; 5 INSERM U-888, Montpellier, France; Institute of Psychiatry at the Federal University of Rio de Janeiro, Brazil

## Abstract

It has been demonstrated that motor coordination of interacting people plays a crucial role in the success of social exchanges. Abnormal movements have been reported during interpersonal interactions of patients suffering from schizophrenia and a motor coordination breakdown could explain this social interaction deficit, which is one of the main and earliest features of the illness. Using the dynamical systems framework, the goal of the current study was (i) to investigate whether social motor coordination is impaired in schizophrenia and (ii) to determine the underlying perceptual or cognitive processes that may be affected. We examined intentional and unintentional social motor coordination in participants oscillating hand-held pendulums from the wrist. The control group consisted of twenty healthy participant pairs while the experimental group consisted of twenty participant pairs that included one participant suffering from schizophrenia. The results showed that unintentional social motor coordination was preserved while intentional social motor coordination was impaired. In intentional coordination, the schizophrenia group displayed coordination patterns that had lower stability and in which the patient never led the coordination. A coupled oscillator model suggests that the schizophrenia group coordination pattern was due to a decrease in the amount of available information together with a delay in information transmission. Our study thus identified relational motor signatures of schizophrenia and opens new perspectives for detecting the illness and improving social interactions of patients.

## Introduction

It has been demonstrated that the interaction at the level of body movements plays a crucial role in the success of social exchanges. Motor coordination of interacting people influences social cognitive functioning, for example, it impacts feelings of connectedness or interpersonal rapport [1]–[4] and communication [5]–[7]. Abnormal movements during social interactions have been reported in patients with schizophrenia, which may explain their interpersonal deficits [8]–[10]. Moreover, such abnormal movements may be the earliest sign of schizophrenia and be used as a marker for earlier diagnostics of this illness that affects almost one percent of the world population [10]–[13]. However, currently no quantitative and objective measures of social motor coordination in schizophrenia have been proposed and the impaired perceptual and cognitive processes underlying it remain largely unknown.

Previous research has shown the relevance of theories and tools of dynamical systems to examine social motor coordination [14]. Such research for instance has demonstrated that the daily interpersonal coordination of arms or postures when two people have verbal exchanges is constrained by the dynamical entrainment processes of coupled oscillators [15]–[18] and that its complexity can be captured over time by one variable, i.e., the relative phasing of the participants' movements. In studies that have evaluated both intentional and unintentional interpersonal coordination, in-phase and anti-phase patterns of motor coordination, characterized by relative phase values of 0° and 180°, are preferentially adopted by visually coupled individuals [18]–[21]. Such patterns occur spontaneously and intermittently when the participants are not instructed to synchronize their movements (i.e., unintentional coordination) and can be stably maintained when the coordination is intentional. For both unintentional and intentional coordination, the stability of these patterns — characterized by the variability of the relative phase — is moderated by the difference between the preferred frequencies of actors' movements with higher stability obtained when their preferred frequencies are close to each other [18], [22]. In addition, their stability depends also on the strength of the perceptual coupling linking two people, which is mediated by how the actors visually attend to the movements of each other [22]–[24].

**Figure 1:**
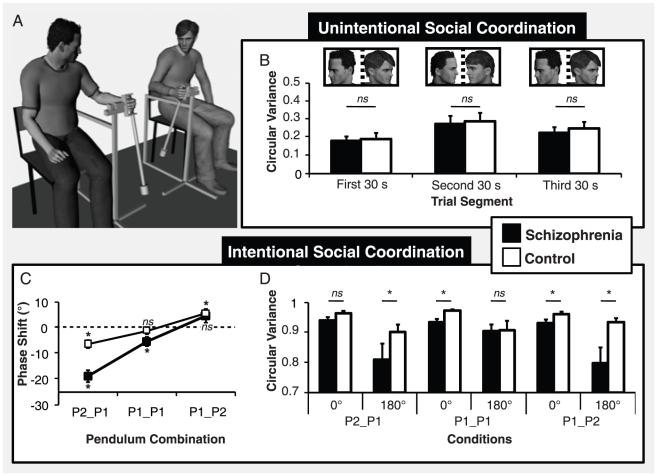
Experimental setup and results for unintentional and intentional social motor coordination. (A) Participants sat on chairs side-by-side while oscillating wrist pendulums. Circular variance of the relative phase for the schizophrenia (black) and control groups (white) as a function of the trial segment for unintentional coordination (B) and of the pendulum combination and the pattern intended for intentional coordination (D). (C) Phase shifts from the intended pattern of coordination as a function of the pendulum combination for the two groups of participants (Asterisks in (C) indicate significant differences from zero). Error bars represent standard error.

Using the dynamical systems methodology, the goal of the current study was to evaluate the dynamics of social motor coordination in people with schizophrenia in order to further characterize their abnormal interpersonal movements and understand the processes affected by the pathology. More specifically, we assumed that the stability of the coordination could be decreased in schizophrenia because of either (i) a modification of their preferred frequency of movement due to their motor disorder [25]–[26] or (ii) a decrease in the strength of the perceptual coupling due to general impairments in attention and visual perception [27]–[30]. To investigate these questions, we examined the unintentional and intentional social motor coordination of individuals with schizophrenia while coordinating the swinging of hand-held pendulums with another individual [15], [18] (see Figure 1A). We compared the coordination produced by twenty patients suffering from schizophrenia (criteria DSM-IV-TR) paired with twenty healthy participants (schizophrenia group) and the coordination produced by twenty pairs of healthy participants (control group). After a practice session in which participants individually swung pendulums, they were tested first in an unintentional coordination task and then in an intentional coordination task.

## Results

To investigate unintentional social coordination, each pair of participants performed three 90 s trials each containing three 30 s segments completed continuously [15], [31]. For each trial, participants were instructed to oscillate their pendulum at a self-selected comfort tempo and maintain it while they were looking (second segment) or not looking (first and third segments) at the oscillatory movements of their partner. The mass of the pendulums was either positioned at the low of the pendulum (P1) or at the middle of the pendulum (P2) to manipulate the self-selected comfort tempo (preferred frequencies) of participants. Each participant pair performed three trials one for each pendulum combination, P2_P1, P1_P1 and P1_P2 and the angular positions of the pendulums of the two participants were recorded. The order of the pendulum combinations was counterbalanced across participant pairs.

To test first whether the preferred frequencies of movements in schizophrenia could be affected and could destabilize social motor coordination, we compared the preferred frequencies of patients and control participants without and before visual interaction (first segment). A repeated-measures 2 (Group)×2 (Pendulum) ANOVA revealed a significant main effect of Pendulum (*F*(1, 19) = 329.71, *p*<.05, η^2^ = 0.95) indicating that in accordance with previous research the preferred frequency of participants was higher when the limbs inertial loadings was less (i.e., the mass of the pendulum was attached at the middle, P2) [15], [18]. However, the analysis did not show any significant effect of Group (*F*(1, 19) = 4.25, *p*>.05, η^2^ = 0.18) or interaction between Group and Pendulum (*F*(1, 19) = 1.60, *p*>.05, η^2^ = 0.08).

To analyze the stability of unintentional social coordination, we computed the circular variance of the relative phase angles between the two times series of participants giving an index of synchronization between 0 and 1 with 1 reflecting a perfect synchronization and 0 reflecting an absence of synchronization [31]–[32]. Higher degrees of coordination were expected during visual interaction (second segment) and when participants used the same pendulums (P1_P1) in view of previous studies [15], [18], [31]. Assuming that schizophrenia creates perceptual or attentional deficits that would decrease the strength of the visual coupling, lower degrees of spontaneous synchronization were expected for the schizophrenia group compared to the control group during the visual interaction (second segment). After a standardization of the circular variance values using a Fisher z transformation, a repeated-measures 2 (Group)×3 (Trial Segment)×3 (Pendulum Combination) ANOVA revealed a significant effect of Trial Segment (*F*(2, 38) = 7.81, *p*<.05, η^2^ = 0.29), of Pendulum Combination (*F*(2, 38) = 8.58, *p*<.05, η^2^ = 0.31) but no significant effect of Group (*F*(1, 19) = 0.36, *p*>.05, η^2^ = 0.02) (see Figure 1B). Further none of the analysis interactions were significant either. As expected, post hoc comparisons (Newman-Keuls) revealed higher degrees of coordination when visual information was available about their partner's movements (second segment) (*p*<.05) and when participants oscillated the same pendulums (P1_P1) (*p*<.05). Although previously reported behavioral deficits in the schizophrenia [27]–[30] led to the expectation that we would see a decrease in unintentional coordination during visual interaction, our results did not show any differences between the schizophrenia group and the control group.

To investigate whether schizophrenia affects intentional social coordination, the same pairs of participants were then instructed to perform together in-phase or anti-phase swinging of their wrist pendulums for 60 s using the pendulum combinations of P2_P1, P1_P1 and P1_P2 [19], [22], [33]. The order of the pendulum combinations was again counterbalanced across participant pairs. As for unintentional coordination, we computed the circular variance of the relative phasing of the two participants' pendulum oscillations to evaluate the stability of the coordination [31]–[32]. After a standardization of the circular variance values using a Fisher z transformation, a repeated-measures 2 (Group)×2 (Pattern)×3 (Pendulum Combination) ANOVA revealed significant effects of Pattern (*F*(1, 19) = 74.84, *p*<.05, η^2^ = 0.80) and Pendulum Combination (*F*(2, 38) = 5.82, *p*<.05, η^2^ = 0.23). These effects support previous research [19], [22], [33] and indicate higher degrees of stability for in-phase coordination and when participants oscillated the same pendulums (P1_P1) (*p*<.05). Moreover, this analysis also revealed a significant effect of Group (*F*(1, 19) = 4.61, *p*<.05, η^2^ = 0.20) and a significant interaction between Pattern, Pendulum Combination and Group (*F*(2, 38) = 4.27, *p*<.05, η^2^ = 0.18). These effects are due to lower stabilities for the schizophrenia group for both in-phase (P1_P1 and P1_P2) (*p*<.05) and anti-phase coordination (P2_P1 and P1_P2) (*p*<.05) (see Figure 1D). These results support our hypothesis that the social motor deficits in schizophrenia could be associated with a decrease of the perceptual coupling strength.

We computed then the phase shift or the deviation of the relative phasing from the intended pattern of 0° or 180° to further explore the stability of the intentional coordination and to determine which participant of the pair led within a cycle [22], [33]. According to the convention used to calculate relative phase, positive phase shifts indicated that the patients in the schizophrenia group led in the coordination and negative phase shifts indicated that patients followed the movements of the other participant. Furthermore, in accordance with previous research showing that the leader of the pair depends on the pendulums used by participants, we expected (i) a phase shift of 0° when participants used the same pendulums (P1_P1) indicating no leader in the dyad, and (ii) a negative phase shift for P2_P1 and a positive phase shift for P1_P2 revealing a leading behavior of the participant swinging the pendulum with the smaller inertial loading (P2) [22], [33]. We also expected (iii) larger phase shifts for P2_P1 and P1_P2 in the anti-phase coordination, which is known to be less stable than in-phase coordination, as well as (iv) larger phase shifts for the Schizophrenia group due to a decrease of the perceptual coupling strength for that group. As expected, a repeated-measures 2 (Group)×2 (Pattern)×3 (Pendulum Combination) ANOVA revealed a significant effect for Pendulum Combination (*F*(2, 38) = 63.46, *p*<.05, η^2^ = 0.77) and a significant interaction between Pattern and Pendulum Combination (*F*(2, 38) = 4.38, *p*<.05, η^2^ = 0.19) indicating, respectively, lower and higher phase shifts than P1_P1 for P2_P1 and P1_P2 (*p*<.05), and a larger negative phase shift in anti-phase than in-phase for P2_P1 (*p*<.05). Interestingly, the analysis showed also a significant effect of Group (*F*(1, 19) = 8.75, *p*<.05, η^2^ = 0.32) and a significant interaction between Group and Pendulum Combination (*F*(2, 38) = 3.61, *p*<.05, η^2^ = 0.16) indicating larger phase shift for P2_P1 for the schizophrenia group (*p*<.05) and no significant differences between the two groups for P1_P1 (*p*>.05) and P1_P2 (*p*>.05) (see Figure 1C). No other effects reached significance. To further examine these results, we performed single sample t-tests contrasting the obtained phase shifts to zero. As expected, the phase shifts of the Control group were significantly different from zero for P2_P1 (*t*(39) = −4.07, *p*<.05) and P1_P2 (*t*(39) = 3.54, *p*<.05) but not significantly different for P1_P1 (*t*(39) = −1.01, *p*>.05) [22], [33]. The phase shifts of the group Schizophrenia were not significantly different from 0° for P1_P2 (*t*(39) = 1.76, *p*>.05) but significantly lower than 0° for both P2_P1 (*t*(39) = −8.11, *p*<.05) and P1_P1(*t*(39) = −2.98, *p*<.05), indicating that patients suffering from schizophrenia never led in the swinging of the pendulums irrespective of the pendulum combination—that is, they did not lead even in those conditions which past research suggests that they should have.

## Discussion

In the current study using the dynamical systems methodology, we investigated social motor coordination in patients suffering from schizophrenia to better characterize and understand the motoric nature of their interpersonal interactions. We examined their unintentional and intentional social motor coordination using a task in which they coordinated the swinging of a pendulum with someone else.

Our results demonstrate that social motor coordination is impaired in schizophrenia. These results extend previous research that indicates impairments of nonverbal behavior in schizophrenia and show that the movements of patients are abnormally coordinated while interacting with another individual [8]–[10], [34]. However, our results demonstrate that only intentional coordination is impaired and that spontaneous, unintentional coordination remains unaffected. This result is in line with previous research that has demonstrated that explicit processing mechanisms are generally more affected than implicit processing mechanisms in schizophrenia. For example, implicit cognitive and emotional processes are relatively preserved in people with schizophrenia compared to explicit processes [35]–[36]. Additionally, such a dissociation has also been reported for impairments of social processes [37], [38].

In the intentional coordination task, our analysis of the relative phasing of the pendulum oscillations showed that the dyads in which a patient was present had a lower stability and that the patient never led in the coordination. These results demonstrate that it is possible to distinguish patients with schizophrenia from control participants by looking at their intentional social motor coordination. Such objective and quantitative differences could possibly be used as a marker of the illness and nicely complement the more subjective evaluations usually performed by clinicians. In line with this expectation is the absence of correlations between the coordination impairments of patients and the severity of their symptoms evaluated by the Positive and Negative Syndrome Scale (PANSS) [39]. Whatever the intended pattern of coordination and the pendulum combination, no correlation between the mean or circular variance of the relative phase and the PANSS Positive, PANSS Negative, PANSS Psychopathology as well as PANSS total were significant for the schizophrenia group (all *ps*>.05). These results suggest that examining intentional social motor coordination of patients might objectively and quantitatively assess abnormal social features that are not captured by the PANSS. Furthermore, because social motor disorders may be the earliest sign of schizophrenia, such marker may allow earlier diagnostics and thus better patient management [10]–[12]. We consider these issues as crucial directions for future research.

We have demonstrated impairments of social motor coordination in schizophrenia but our study also aimed to understand the impaired underlying processes. We initially assumed that impairments could be the consequence of attention and visual perception deficits that may affect the visuo-motor processes underlying the maintenance of coordination. More specifically, in terms of coupled oscillators system accounting for social rhythmic coordination, we predicted a decrease of the coupling strength, which corresponds to a lower sensitivity to the movements of the other. We found a decrease in the stability of intentional coordination in the schizophrenia group that is compatible with this hypothesis [22]–[23]. However, a coupling strength account is not sufficient in explaining the pattern of results observed for the phase shift measure. A lower coupling strength for the schizophrenic group would predict an exaggerated phase shift relative to the control group for both conditions in which the participants swung different pendulums (more positive than control for P1_P2 and more negative than control for P2_P1) but zero phase shift when the pendulums were identical (P1_P1) [22], [33]. However, as can be seen in Figure 1C, the schizophrenic group phase shift is characterized by an absolute lowering which indicates that they tended not to lead the coordination.

Slower preferred movement frequencies in schizophrenia could explain that patients suffering from schizophrenia never led the coordination [22], [33]; however, the analysis performed on preferred frequencies above did not show any significant differences between the preferred frequencies of the two groups. A more convincing explanation of this result is that patients with schizophrenia have a delay in processing information for visuo-motor control. In addition to their contribution in decreasing coupling strength, attention and visual perception deficits may also impair information transmission and be understood as an increase of the time delay in the coupling function [40]. This hypothesis is supported by previous research showing slower reaction times in schizophrenia [41] and more specifically by anatomical correlates such as a degradation of the degree of myelination [42].

To further explore these hypotheses and determine how a decrease of the strength or/and a delay in the information transmission of patients could explain their impairments of intentional social motor coordination, we compared our phase shift results with those obtained in simulations of a coupled oscillator model that has been used in the past for understanding social motor coordination [43]–[44]. The model utilizes a non-linear coupling of two limit-cycle oscillators:
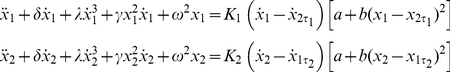
(1)where *x*_1_ and *x*_2_ represent the positions of the two oscillators and the dot notation represents derivative with respect to time. The left side of the equations represents the limit cycle dynamics of each oscillator determined by linear stiffness parameter (ω) and damping parameters (*δ*, *λ*, *γ*) and the right side represents the coupling function determined by parameters *a* and *b*. To simulate impairments due to schizophrenia, we added the parameters *K*_1_ and *K*_2_ corresponding to the coupling strengths of the oscillators 1 and 2, 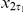
and 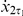 corresponding to the position and the velocity of the oscillator 2 at a previous time point 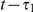 and the parameters 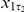
and 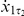
corresponding to the position and the velocity of the oscillator 1 at a previous time point 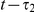.

**Figure 2:**
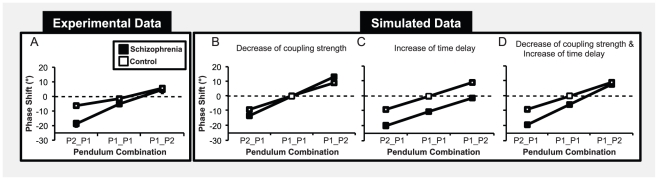
Phase shifts from the intended pattern of coordination as a function of the pendulum combination for the schizophrenia (black) and control groups (white). Phase shifts obtained experimentally (A) and in the simulations with a decrease of the coupling strength (B), with an increase of the time delay (C) and with both of them (D).

A decrease of the strength and an increase of the delay in the coupling function of patients were tested first separately and then combined to examine whether they could capture experimental results of the schizophrenia group. We used the parameters *K* = 1.6 and τ = 0 to simulate the behavior of control participants and the parameters *K* = 0.6 and τ = 3 to simulate impairments of individuals with schizophrenia. Such time delay (τ = 3) represented 1% of the simulated period of P1 and 1% of the experimental period of P1 represented an increase of 75 ms compared to control participants corresponding to plausible physiological delays [45]. The experimental and simulated phase shifts results are presented in the Figure 2. For the simulated data, the phase shifts in white represent the results obtained for the simulated coordination of the control group (τ_1_ = τ_2_ = 0, *K*_1_ = *K*_2_ = 1.6) and in black of the schizophrenia group with a decrease of the coupling strength (τ_1_ = τ_2_ = 0, *K*_1_ = 1.6, *K*_2_ = 0.6) (Figure 2B), with an increase of the time delay (τ_1_ = 0, τ_2_ = 3, *K*_1_ = *K*_2_ = 1.6) (Figure 2C) and with both of a decrease of the coupling strength and an increase of the time delay (τ_1_ = 0, τ_2_ = 3, *K*_1_ = 1.6, *K*_2_ = 0.6) (Figure 2D).

As depicted in Figure 2B, a decrease of the coupling strength in schizophrenia, as initially assumed, cannot explain that patients suffering from schizophrenia never led the coordination. An increase in time delay can predict that patients never led the coordination but cannot explain that the two groups have a similar phase shift for the pendulum combination P1_P2 (Figure 2C). Figure 2D shows that the two parameterization, when combined, capture experimental phase shifts obtained for the schizophrenia group suggesting that intentional social motor coordination impairments results from both a decrease of the strength and an increase of the time delay in the coupling function of patients. Moreover, our results suggest that the absence of difference between the two groups of participants for the pendulum combination P1_P2 did not result from an absence of deficit of patients in this experimental condition, but rather was a consequence of the two impaired processes acting in opposite direction.

Although we have shown that increasing the delay and decreasing the strength in visuo-motor control capture the intentional coordination pattern of the schizophrenic group, further explorations may improve the understanding of the processes involved. Investigating whether such disorders are specific to social visual coordination or to visual coordination in general is an important research direction. In fact, modifications of intentional coordination in schizophrenia could be due to impairments of mechanisms mediating both visual and social visual coordination or may be specific to mechanisms mediating just social visual coordination. It has been demonstrated that patients suffering from schizophrenia have impairments of visual perception of biological motion that can be distinguished from their general visual perception deficits [46]. More particularly, mirror neuron system disorders, which corresponds to the brain area that may also moderate social motor coordination [47]–[48], may play a crucial role in such a deficit and in social interactions deficits in general [49]–[50].

We have modeled the impairments of schizophrenia patients here by changes at the level of the coupling function and not changes in the natural dynamics of the individual oscillators because our experimental results did not show differences between the preferred frequencies of patients and control participants. Although we did not find differences, previous research has reported slowness in movements of patients suffering from schizophrenia [25]–[26]. Such effects may depend on the movement performed and our lack of an effect may be specific to the wrist pendulum task in which the inertial loading of pendulums that may have obscured the slowness of patients. It is possible that using different tasks (e.g., coordination of postural sways [16], [20]), deficits of the movement itself as well as at the level of the visuo-motor control may be observed. These questions encourage explorations to further determine the different origins of impaired coordination of schizophrenia patients while interacting with other people.

More generally, by examining the coordination dynamics of swinging of handheld pendulums, this experiment demonstrates that interpersonal coordination is impaired in schizophrenia without explicitly showing the consequences of such modifications on everyday social interactions of patients. It is however important to note that the coordination dynamics observed in this laboratory task reflect the ones observed for example between hand, arm or postural movements in more ecological situations, which have been demonstrated as directly influencing the success of our social interactions [1]–[7], [14]. Such coordination moderates the social cognitive functioning of interacting people by impacting for instance their feelings of connectedness and interpersonal rapport [1]–[4], or the efficiency of their communication [5]–[7]. In line with these past results, the current study shows how a motor coordination disorder may be an important factor in the everyday social interaction deficits exhibited by patients suffering from schizophrenia.

Therefore, in addition to future research that aims at a further understanding of how schizophrenia affects the processes underlying social motor coordination, it will be important to develop rehabilitation protocols that can help improve the social motor coordination of patients. Although no investigations to our knowledge have yet explored the learning and rehabilitation of social motor coordination, previous research that has examined motor learning may provide interesting research directions [51]–[53]. For example, it has been demonstrated that motor learning can be made more efficient with real-time feedback [53]–[55]. Accordingly, future therapeutic protocols using such feedback may help to improve the motor coordination in general and social motor coordination in particular of patients, and thus, increase their ability to interact with other people in everyday life.

In conclusion, our study provided clear evidence of social motor coordination impairments in schizophrenia that may help us understand their social deficits observed in everyday interpersonal interactions. Our results demonstrate differences between schizophrenic and control dyads in an intentional coordination task—a task that may be used by clinicians as a marker of schizophrenia for better diagnostics. In addition, our dynamical modeling of the results revealed that such pathological intentional coordination may be due to a decrease in the amount of information as well as a delay in the information transmitted about movements of other people. Finally, such findings may lead to the development of rehabilitation protocols improving social motor coordination and successful social exchanges of patients.

## Methods

### Ethics statement

All participants provided written informed consent prior the experiment approved by the local Ethics Committee (CPP Sud Méditérannée III, Montpellier, France, AFSSAPS 2009-A00513-54 24, 07/22/2009) conforming to the Declaration of Helsinki. A clinician who was experienced in the evaluation of mental illness assessed by a direct examination of participants, their understanding of all the procedures and capacity to consent. As proposed by Palmer et al. [56] to assess capacity to consent, each participant was asked three questions about the hypothetical study: (1) “What is the purpose of the study?” (2) “What are the risks?” and (3) “What are the benefits?”. The participants were included in the study only if they had the full capacity to consent.

### Participants

Twenty outpatients who fulfilled the DSM-IV-TR criteria for schizophrenia participated in the study [57]. All patients were in the stable phase of the illness according to the current treating psychiatrist and were receiving antipsychotic medication at the time of their participation. Severity of symptoms was evaluated using the Positive and Negative Syndrome Scale (PANSS) [39] (see Table 1). The twenty patients were matched with twenty *matched control participants* for age, sex, education and premorbid IQ, as estimated by the French adaptation of the National Adult Reading Test (fNART) [58] (all *ps*>.05). During the experiment, the patients were randomly paired with *control participants 1* to compose the schizophrenia group and *matched control participants* were paired with *control participants 2* to compose the control group. *Control participants 1* and *control participants 2* were matched for age, sex, education and premorbid IQ (all *ps*>.05). All participants had normal or corrected-to-normal vision.

**Table 1:** Mean ± standard deviation of demographic characteristics of participants.

	Matching 1				Matching 2			
	Patients (n = 20)	Matched Control (n = 20)	*T/χ^2^*	*P*	Control 1 (n = 20)	Control 2 (n = 20)	*T/χ^2^*	*P*
Age (years)	38.15±10.19	35.60±14.74	.64^a^	.53	23.70±4.94	24.65±5.65	−0.57^a^	.57
Sex (Male/Female), *n*	13/7	7/13	2.5^b^	.11	8/12	10/10	.10^b^	.75
Education (years)	10.55±2.21	11.45±2.06	−1.33^a^	.19	15.20±1.79	15.90±2.02	−1.16^a^	.25
Premorbid IQ (f-NART)	103±8.12	107±9.03	−1.41^a^	.17	110±8.70	106±7.04	1.50^a^	.14
PANSS Positive	13.75±3.65							
PANSS Negative	21.05±6.01							
PANSS Psychopathology	36.20±8.34							
PANSS Total	71.00±14.80							

PANSS: positive and negative syndrome scale; IQ: Intellectual Quotient; f-NART: French version of the National Adult Reading Test.

a Independent-samples T-test.

b Chi-square test.

### Apparatus

Participants sat on chairs approximately 1 m from one another facing in the same direction and swung wrist pendulums attached to a structure that allowed only movements from front to back. The swinging of the pendulums made no noise that could be used as a cue for coordination. The length of the two pendulums was 60 cm and a mass of 150 g was attached either at the bottom or at the middle to constitute the pendulums P1 and P2, respectively. The preferred movement frequency of participants was 0.80 Hz for pendulum P1 and 0.95 Hz for pendulum P2 (averaged across participants in the first segments of unintentional coordination trials). Two potentiometers measured the angular displacements of pendulums during the trials at a sampling rate of 50 Hz.

### Design and Procedure

Upon arrival, participants were informed that the experiment was investigating rhythmic movements with wrist pendulums. Participants then performed practice trials individually, in which they were instructed to hold the pendulum firmly in their hand and to swing it at their own self-selected tempo, a tempo they found comfortable and could maintain for an extended period of time. Patients and *matched control participants* oscillated pendulums with their right hand and *control participants 1* and *2* oscillated pendulums with their left hand. After the practice session with the pendulums P1 and P2, participants were tested together for unintentional social coordination.

Participants were first tested for unintentional social motor coordination. They completed three 90 s trials one for each pendulum combination P2_P1, P1_P1 and P1_P2, corresponding respectively to the pendulums used by *control participants 1* and patients for the schizophrenia group and *control participants 2* and *matched control participants* for the control group. Each trial contained three 30 s segments that were delimited by an auditory stimulus. Participants were instructed to swing pendulum at the same self-selected tempo whether they were looking (second segment) or not looking (first and third segments) at the movements of their partner [15], [18], [31]. During the first and third segments, participants were instructed to focus on crosses that were positioned on the wall in the opposite direction of the other participant. Participants were reminded just prior to each trial to swing the pendulum at their own self-selected tempo and to maintain that tempo throughout the trial.

Participant pairs were then tested for intentional social motor coordination. They were informed that they would be required to coordinate their movements together in an in-phase or anti-phase mode for 60 s [19], [22], [33]. After the instructions, each pair performed one practice trial for in-phase and one for anti-phase. Participants performed three trials for each pattern of coordination with the three different pendulum combinations (P2_P1, P1_P1 and P1_P2). The order of the trials was counterbalanced across participants and the beginning of each trial was initiated with an auditory stimulus.

### Coordination Analysis

The first five seconds of each intentional trial and segment of unintentional trials were discarded to avoid transient behavior. The times series of participants were low-pass filtered using a 10 Hz Butterworth filter. The frequency of oscillation during the first segment of unintentional coordination was computed as the inverse of the average time between the points of maximum angular extension as defined by the maxima of time series. The continuous relative phase between the two angular positions of pendulums was computed using the Hilbert Transform [59]. From the computed relative phase time series, we calculated the circular variance of the relative phase giving an index of synchronization between 0 (no synchronization) and 1 (perfect synchronization) and the phase shift from the intended pattern of coordination for intentional coordination [31]–[32]. Positive phase shifts indicated that patients led the coordination and negative phase shifts indicated that patients followed the movements of the other participant. We used analysis of variance (ANOVA) to examine the influence of schizophrenia on the different variables and Newman-Keuls post-hoc tests as necessary to determine the nature of the effects.

### Simulated Coordination

We simulated Eq. (1) to test whether a decrease of the strength or/and an increase of the time delay in the coupling function captured patients' impairments of intentional social motor coordination [42]–[43]. For all simulations, the parameters were set as *δ* = −0.7, λ = *γ* = 1, *a* = −2, *b* = 2, *ω* = 6.25 for the pendulum P1 and ω = 6.30 for the pendulum P2. We used the coupling strength *K* = 1.6 and delay τ = 0 to simulate the behavior of control participants and *K* = 0.6 and τ = 3 to simulate impairments of patients suffering from schizophrenia. Only in-phase coordination was simulated because our results did not show an effect of the pattern of coordination for the phase shift modifications. Before every simulation, one cycle of each oscillator without coupling (*K*_1, 2_ = 0) was computed and the last samples of each were then aligned to constitute the first sample of the simulation. Such procedure was necessary to obtain initial conditions 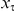 and 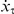 at a previous time point 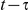 for the simulation when τ was higher than 0. The numerical integrations used an Euler-Maruyama method with a time step of 0.02, a path length of 9000 points for each simulation and we computed phase shifts only using the last 3000 points (around 60 cycles) to avoid transient state. The analysis to compute the relative phase and the phase shift from the intended pattern of coordination was the same as for the experimental data.
